# Health and medical research for all: The challenge remains open

**DOI:** 10.1371/journal.pmed.1002970

**Published:** 2019-10-31

**Authors:** The PLOS Medicine Editors

**Affiliations:** Public Library of Science, San Francisco, California, United States of America and Cambridge, United Kingdom

## Abstract

On our 15th anniversary, the PLOS Medicine editors discuss progress in open access, medical publishing and the journal's mission over the years

Fifteen years ago in October, *PLOS Medicine* was launched with the intent to reinvent the medical journal. Our mission to serve as the leading open-access medical journal is unchanged and remains, we believe, as pressing and essential now as it was at launch. In this Editorial we highlight priorities for the journal in meeting the needs and expectations of clinicians and researchers in the coming years. We also reflect on the evolution of some of the major medical and health challenges affecting the world’s people since our tenth anniversary [1] and discuss some recognizable changes in thinking and approaches in health research, clinical practice and medical publishing. We are also taking this opportunity, along with selected members of our Editorial Board, to highlight our favorite research and commentary articles in a series of blog posts at *Speaking of Medicine* (https://blogs.plos.org/speakingofmedicine/).

At the global level, recognition of the urgent need for concerted action towards the health needs of the whole global population—and alongside it efficient open-access publication of and debate about research findings to speed the pace of progress—has never been clearer. The inextricable relationship between health and development has been detailed in the progression from Millennium Development Goals post-2015 to the Sustainable Development Goals (SDGs), a set of 17 aspirational areas for investment and monitoring, all with explicit targets and indicators, running during the period 2015 to 2030 [2]. SDG3 focuses on health and well-being, and even at this relatively early stage in the SDG process, there are examples of serious challenges in realizing health goals in the key population of children in many parts of the world, such as [3]. Achievement of universal health coverage is a key element of SDG3, a theme that *PLOS Medicine* aims to support and scrutinize along with other challenges in global health, as illustrated in a recent Collection on health system quality assembled together with *PLOS ONE* [4].

With disease prevention paramount in modern health care, infectious diseases relentlessly probe the capacity of health systems to protect all individuals against emerging and recognized pathogens, irrespective of countries’ wealth or stage of development. This challenge is graphically shown in the current Ebola outbreak in the Democratic Republic of the Congo, which at the time of writing has claimed an estimated 3157 cases and 2108 deaths [5]. Despite widespread alarm at the lackadaisical response to the earlier West African outbreak of 2013 to 2016, the heavy toll of mortality and ill health in the latest outbreak reminds us that research on protection of and treatment for people with emerging infectious diseases is neglected at our peril. With rising global temperatures due to climate change causing a potential domino effect, ocean expansion, spatial reorganisation of disease vectors, and displacement of communities are among the biggest health challenges of our time [6]. *PLOS Medicine* aims to play a crucial role in the dissemination of research in these areas as indicated by our 2017 Special Issue on Climate Change and Health [7] as well as our current Special Issue focussing on Refugee and Migrant Health [8].

Turning to familiar pathogens, the ongoing struggle with measles has been well publicized, not only in low- and middle-income countries in which vaccination programmes are incomplete, but also in the populations of high-income countries where destructive elements have contrived to interfere with achievement of proper vaccination coverage. The campaigns to undermine public confidence (often in tight-knit communities vulnerable to sharp decreases in herd immunity) in vaccine safety and efficacy well illustrates the advocacy role of medical journals and other responsible media sources in countering health misinformation [9].

In clinical research, there is a continuing tension between the well-honed tenets of evidence-based medicine—that interventions should be proven in rigorous randomized controlled trials in relevant populations, for example—and the advent of precision medicine, in which it is anticipated that treatments will be tailored to the individual patient or disease. Early licensing of promising therapeutic agents based on intermediate-stage trial results is now expected in high-income countries, supported by formal requirements for ongoing evaluation of clinical and safety outcomes. In this environment, comparative effectiveness research is likely to be increasingly important, as it is in evaluation of the effectiveness of generic and brand-name medications in practice, for example [10]. As indicated in our recent Special Issue devoted to research on machine learning in health and biomedicine, *PLOS Medicine* will also seek to further progress in medicine by focusing on research aiming to use computational techniques to aid diagnostic, prognostic and treatment decisions [11].

Five years ago, we commented that “open access to medical research has become more complicated than just choosing an idealistic new journal over regressive old ones”, referring to the labyrinth of hybrid subscription and article processing charge publishing models that exists, often disingenuously crafted so as to protect the business models of for-profit publishers. This unhelpful situation prevails today and prevents access in a fashion that could honestly be described as “open”, for many readers, to a large proportion of newly published research papers. We hope that the ongoing initiative Plan S—supported by the research funder group cOAlition S—will be able to resolve this issue by 2021 [12,13].

As the scientific publishing landscape continues to evolve, *PLOS Medicine* remains in step with the implementation of strategies that facilitate rapid and efficient communication of medical research. Along with other PLOS journals, *PLOS Medicine* has recently begun to welcome submission of preprints uploaded to the server medRxiv, for example [14]. This development is intended to provide greater transparency in the reporting and assessment of research, consistent with earlier successful initiatives aiming to add clarity to the process of clinical trial registration and reporting. Beginning early this year, PLOS journals now offer authors the opportunity to publish the peer review history of articles, an initiative that aims to increase transparency of the peer review process and reduce waste where articles may undergo consideration at more than one journal. Consistent with the ambition of PLOS to streamline and democratize the processes of scientific and medical publishing, further innovations are anticipated, for instance in providing recognition to peer reviewers, whose constructive contributions to scientific and medical publications are woefully under-recognized at present. We welcome your comments and suggestions and look forward to readers contributing as peer reviewers as well as submitting their research and discussion papers to *PLOS Medicine*.
